# In-House, Open-Source 3D-Software-Based, CAD/CAM-Planned Mandibular Reconstructions in 20 Consecutive Free Fibula Flap Cases: An Explorative Cross-Sectional Study With Three-Dimensional Performance Analysis

**DOI:** 10.3389/fonc.2021.731336

**Published:** 2021-09-24

**Authors:** Lucas M. Ritschl, Paul Kilbertus, Florian D. Grill, Matthias Schwarz, Jochen Weitz, Markus Nieberler, Klaus-Dietrich Wolff, Andreas M. Fichter

**Affiliations:** ^1^ Department of Oral and Maxillofacial Surgery, School of Medicine, Technical University of Munich, Klinikum rechts der Isar, Munich, Germany; ^2^ Department of Oral and Maxillofacial Surgery, Josefinum, Augsburg and Private Practice Oral and Maxillofacial Surgery im Pferseepark, Augsburg, Germany

**Keywords:** in-house CAD/CAM planning, 3D printing, mandibular reconstruction, free fibula flap, open-source software

## Abstract

**Background:**

Mandibular reconstruction is conventionally performed freehand, CAD/CAM-assisted, or by using partially adjustable resection aids. CAD/CAM-assisted reconstructions are usually done in cooperation with osteosynthesis manufacturers, which entails additional costs and longer lead time. The purpose of this study is to analyze an in-house, open-source software-based solution for virtual planning.

**Methods and Materials:**

All consecutive cases between January 2019 and April 2021 that underwent in-house, software-based (Blender) mandibular reconstruction with a free fibula flap (FFF) were included in this cross-sectional study. The pre- and postoperative Digital Imaging and Com munications in Medicine (DICOM) data were converted to standard tessellation language (STL) files. In addition to documenting general information (sex, age, indication for surgery, extent of resection, number of segments, duration of surgery, and ischemia time), conventional measurements and three-dimensional analysis methods (root mean square error [RMSE], mean surface distance [MSD], and Hausdorff distance [HD]) were used.

**Results:**

Twenty consecutive cases were enrolled. Three-dimensional analysis of preoperative and virtually planned neomandibula models was associated with a median RMSE of 1.4 (0.4–7.2), MSD of 0.3 (-0.1–2.9), and HD of 0.7 (0.1–3.1). Three-dimensional comparison of preoperative and postoperative models showed a median RMSE of 2.2 (1.5–11.1), MSD of 0.5 (-0.6–6.1), and HD of 1.5 (1.1–6.5) and the differences were significantly different for RMSE (*p* < 0.001) and HD (*p* < 0.001). The difference was not significantly different for MSD (*p* = 0.554). Three-dimensional analysis of virtual and postoperative models had a median RMSE of 2.3 (1.3–10.7), MSD of -0.1 (-1.0–5.6), and HD of 1.7 (0.1–5.9).

**Conclusions:**

Open-source software-based in-house planning is a feasible, inexpensive, and fast method that enables accurate reconstructions. Additionally, it is excellent for teaching purposes.

## Introduction

The application of computer-aided design and computer-aided manufacturing (CAD/CAM) technology in primary and secondary mandibular reconstruction with the free fibula flap (FFF) following ablative surgery is considered to be state of the art nowadays. Several studies have proven its benefits and superiority in terms of operating time, ischemic time, symmetry, bony consolidation, and function (1–4), which may result in a positive cost–benefit balance sheet (5, 6) as well as better functional and aesthetic results (7–9).

After increasing standardization of the surgical processes and the integration of virtual planning processes in the last decade, the trend continues toward cost reduction, as patient-specific cutting guides and osteosynthesis plates are usually offered and produced by various osteosynthesis manufacturers and may even not have led to overall cost reduction (10). On the one hand, this necessitates a functioning infrastructure with nationwide coverage by these companies, and on the other hand, the effort expended entails additional costs for the surgical department and ultimately the healthcare system (10). In addition, the dependence on the industry reduces flexibility of planning timing and, depending on the complexity of the case, requires a lead time of at least seven to ten working days, during which one to three web meetings are held to discuss the planning and its implementation. In this context, two developments can be observed in the daily routine and more recent literature: first, the establishment of low-cost solutions for the in-house production of cutting guides using open-source software and in-house printers (11–14) and, second, the use of partially adjustable resection aids such as the ReconGuide (KLS Martin Group; Gebrüder Martin GmbH & Co. KG; Tuttlingen, Germany) and the MUC-Jig (15, 16).

The purpose of this study was to evaluate our workflow and results of in-house-planned mandibular reconstructions with the FFF and to describe potential pitfalls and solutions for a wider application of this versatile opportunity.

## Materials and Methods

### Ethical Statement and Enrolled Patients

All clinical investigations were conducted according to the principles expressed in the Declaration of Helsinki. The exploratory cross-sectional study of a historical cohort was approved by the institutional ethics committee of the Technical University of Munich, Klinikum rechts der Isar (Approval number: 326/21 S-EB).

**Figure 1:**
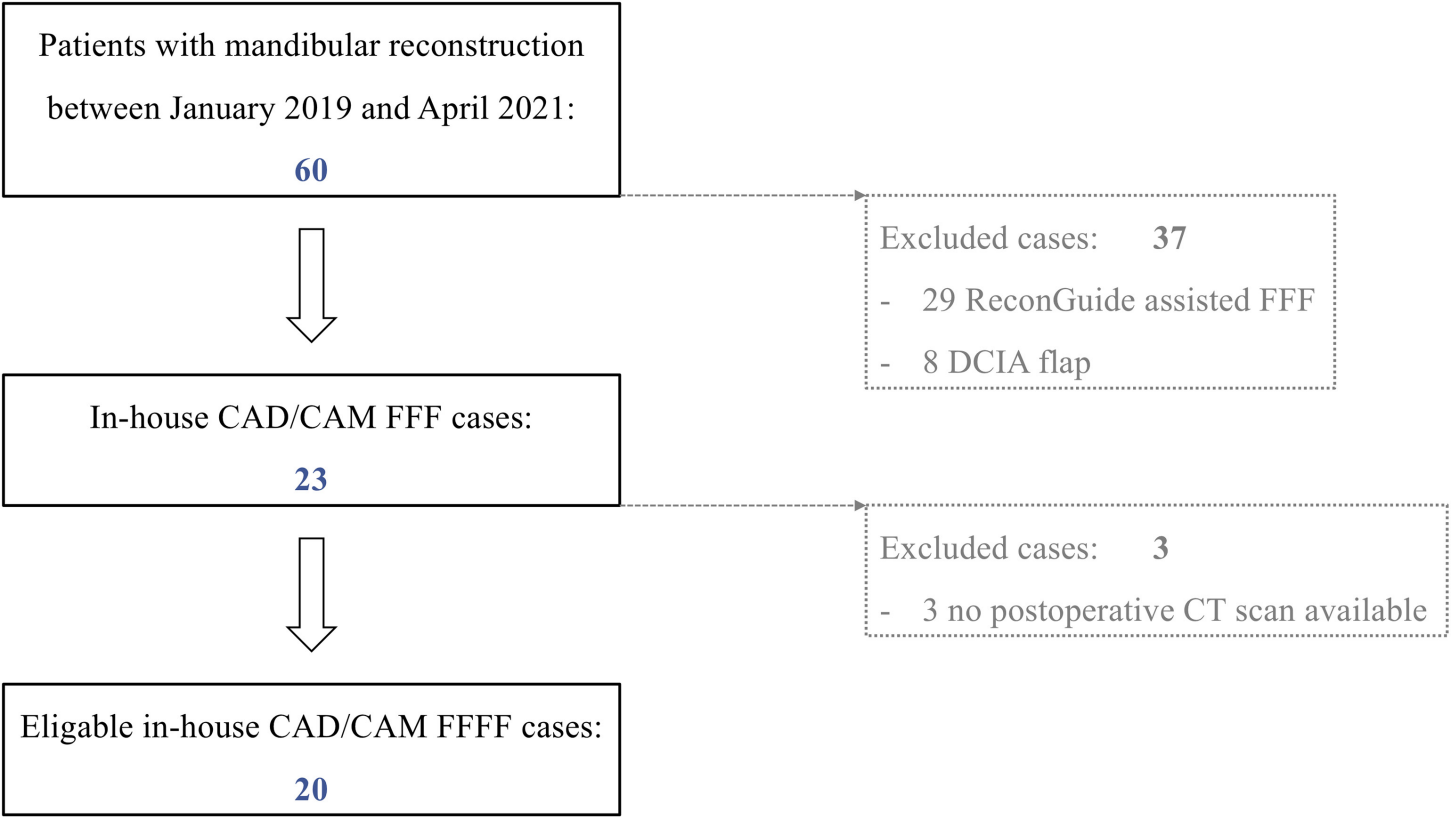
Patient enrollment protocol of this exploratory cross-sectional study of a historical cohort. (FFF, free fibula flap; DCIA, microvascular iliac crest flap, deep circumflex iliac artery).

All patients who underwent mandibular resection and a reconstruction with an in-house-planned CAD/CAM FFF in our department for a benign or malignant disease between January 2019 and May 2021 were included. Patients without a postoperative computed tomography (CT) scan or with any otherwise planned and performed mandibular reconstruction (for example the use of partially adjustable resection aids or other microvascular bone flap) within this observation period were excluded (**Figure 1**). Data collection included: gender, age, indication for mandibular reconstruction, extent of resection according to Brown et al. (I–IV) (17), number of fibular segments, duration of surgery [min], ischemia time [min], and estimated resin volume and printing duration per case. Ischemia time was defined as the interval between ligation of the pedicle, mandibular reconstruction with completed osteosynthesis and opening of the vessel clamp following microvascular anastomoses.

All enrolled FFF cases were preoperatively planned and flaps were harvested by either the first- or last-named author (LMR, AMF), using the lateral approach (18) and using templates printed in-house (cutting guides and repositioning aids).

### Blender-Based In-House CAD/CAM Planning

The digital workflow for the production of the in-house-planned and printed cutting and reconstruction guides complies with the general principles of CAD/CAM-assisted techniques as described elsewhere in detail (1, 19–21).

**Figure 2:**
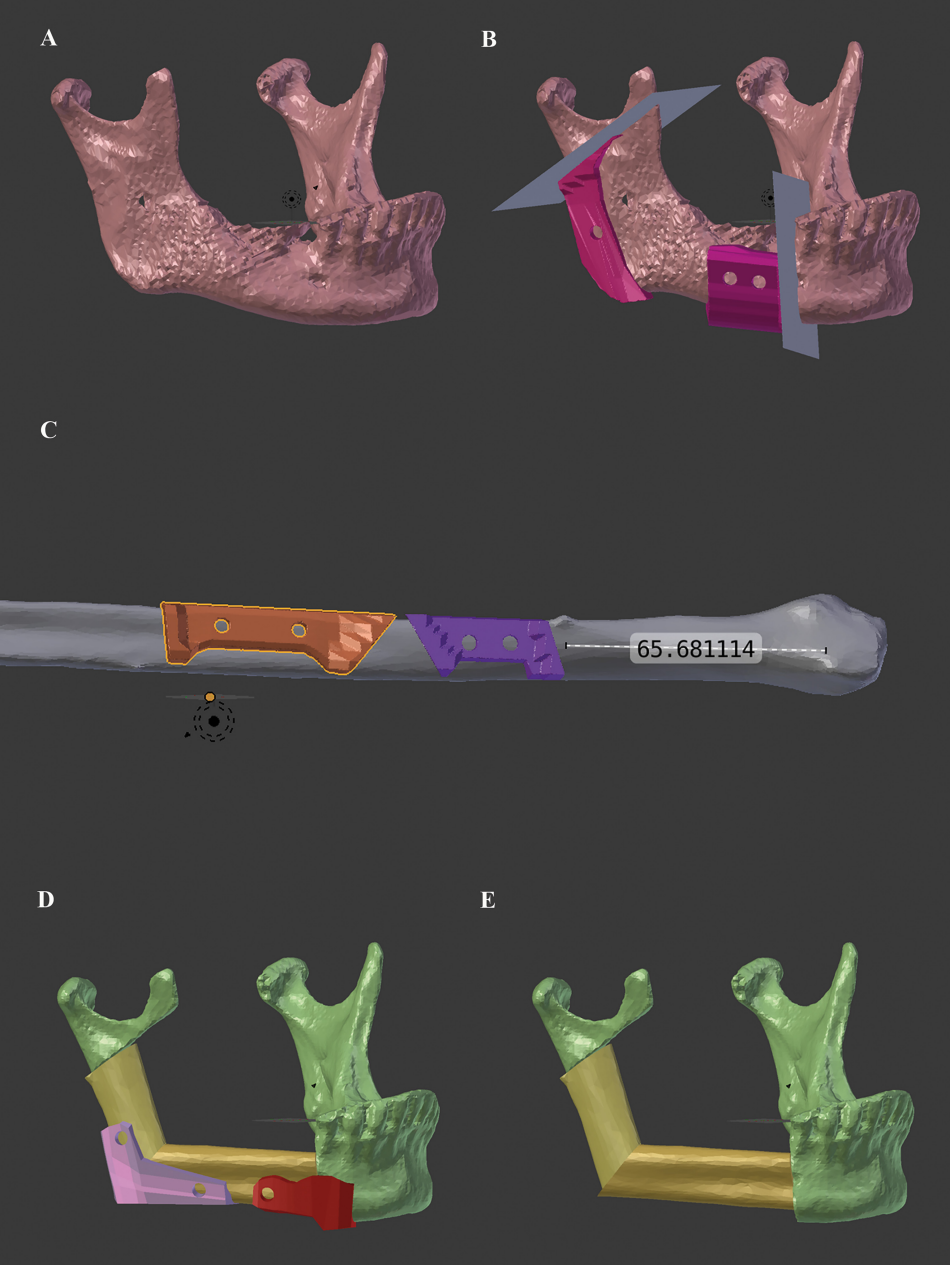
Workflow for in-house Blender-based (Blender^®^ Version 2.79; Blender Foundation and Institute; Amsterdam, Netherlands) planning and design of corresponding mandibular and fibular cutting guides and repositioning aids for mandibular reconstruction with the free fibula flap. **(A)** preoperative mandibular situation, **(B)** simulated resection planes and designed mandibular cutting guides, **(C)** corresponding fibular cutting guides, **(D)** repositioning aids (at angle and neomandibular/mandibula body junction), and **(E)** final virtually reconstructed mandible.

Available pre- and postoperative CT scans of enrolled patients were collected. Corresponding Digital Imaging and Communications in Medicine (DICOM) data sets of the CT scans were anonymized and converted to corresponding standard tessellation language (STL) files using Mimics^®^ software (Mimics^®^ 17.0, Materialise; Leuven, Belgium). The open-source software solution Blender (Blender^®^ Version 2.79; Blender Foundation and Institute; Amsterdam, Netherlands) was used for the in-house computer planning and design of corresponding mandibular and fibular cutting guides and repositioning aids (**Figure 2**). No validation or comparison of the Blender-based planning with another software was performed.

The purely Blender-based planning procedure contained following key steps (**Supplementary Data**): First, the preoperative STL file of the mandible was imported to the Blender software and positioned in three-dimensional space (“*Object_Transform_Geometry to Origin*”). Then the necessary cutting planes were set and aligned as surgically required (paramedian, corpus, ramus, etc.) (“*Create_Cube*” → transform and manipulate in *object* and *edit mode*). Now, depending on the number of necessary fibula segments, the STL file of the fibula was imported and superimposed onto the expected mandibular defect according to the functional and reconstructive requirements. After simulation of the reconstructive result (= neomandibula) and final adjustment of the fibula segment positions, the corresponding cutting guides for the mandible and the fibula, as well as the repositioning aids for the final neomandibula, were designed (“*Create_Cube*” → transformation and manipulation in *object* and *edit mode*, and “*Add Modifier_Boolean_Difference*”). Repositioning aids were only created for the junction between neomandibula body and original mandibular as well as for the neomandibular angle. No repositioning aids were designed for the ramus/condylar junction due to lack of space. In this region, pre-bent miniplates were the only aid to transfer the virtual plan.

In addition, holes with a diameter of 4.2 mm were integrated to incorporate drill sleeves for safe intraoperative temporary fixation (KLS Martin Group; Gebrüder Martin GmbH & Co. KG; Tuttlingen, Germany).

The cutting guides and repositioning aids were printed in-house with a Form 2 stereolithographic printer (Form 2, Formlabs; USA) using a Class 1 autoclavable, biocompatible photopolymer resin (Dental SG, FLDGOR01; Formlabs; USA) with a layer thickness of 50 µm. The post-processing of the printed geometries was carried out according to the manufacturer’s instructions.

### Conventional Measurements and Three-Dimensional Analysis of Postoperative Results

Postoperative analysis of the surgical results included conventional measurements and three-dimensional surface matching methods. The conventional measurements included the following distances: horizontal distance condylar head–condylar head (head–head) medial (1), lateral (2), and the condylar angles left (3) and right (4) according to Ueki et al. (22).

**Figure 3:**
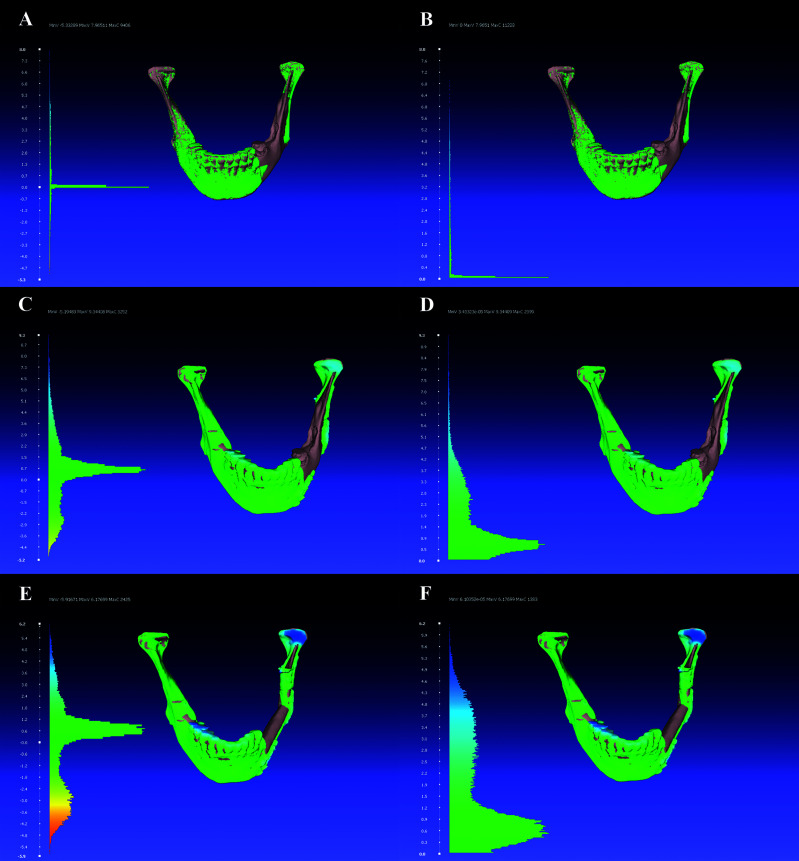
Three-dimensional analyses were done with the open-source software MeshLab (MeshLab_64bit_fp v2020.12) showing root mean square error [RMSE, (mm)] in the left column and Hausdorff distance [HD, (voxel)] in the right column: **(A, B)** preoperative *vs*. virtual (pre-virt) model, **(C, D)** preoperative *vs*. postoperative (pre-post), and **(E, F)** virtual model *vs*. postoperative (virt-post).

After the segmented pre- and postoperative mandibles and the virtual model were six-point-aligned, the following three three-dimensional parameters were determined for the comparison of three possible constellations [preoperative *vs*. virtual model (pre-virt); preoperative *vs*. postoperative model (pre-post); virtual model *vs*. postoperative (virt-post)]: root mean square error [RMSE, [mm)], mean surface distance [MSD, (mm)], and Hausdorff distance [HD, (voxel)] (23–25) (**Figure 3**). All conventional and three-dimensional analyses were performed using the open-source software MeshLab (MeshLab_64bit_fp v2020.12) and Blender.

All measurements were performed independently by two investigators (PK and FDG). All analyses were performed twice; the second round of analysis was performed at least seven to fourteen days later to minimize a habitual landmark setting and six-point alignment (26).

### Statistical Analysis

The intraclass correlation (ICC) coefficient (Cohen’s kappa = κ) was calculated to determine the intra- and interrater reliability and consistency of measurements performed by two raters applying a two-way mixed model. For the analysis of pre- and postoperative differences of the conventional parameters the Wilcoxon signed-rank test was used. For the differences of RMSE, MSD, and HD between pre-virt *vs*. pre-post models uni- and multivariate regression analyses were performed.

All statistical tests were performed on an exploratory two-sided 5% significance level. No adjustments were made for multiple testing. Analysis was done with IBM SPSS 24 for Mac software (IBM Corp, Armonk; New York, United States).

## Results

### General Information and Descriptive Statistics

Twenty patients (9 female, 11 male) met the inclusion criteria (**Figure 1**). Age, indication for surgery, distribution of mandibular defect class according to Brown et al. (17), and the distribution of corresponding number of segments are shown in **Table 1**.

**Table 1:** Overview of enrolled patients with regard to registered parameters: gender, age, indication for surgery, mandibular defect class according to Brown et al. (17), number of segments.

Parameters	n (%)	
Gender female/male	9/11	
Age median (range)	55.5 (23–79)	
Indication	OSCC	5 (25%)
	Secondary reconstruction	5 (25%)
	ORN	4 (20%)
	Ameloblastoma	2 (10%)
	Osteomyelitis	2 (10%)
	MRONJ	1 (5%)
	Ameloblastic fibrosarcoma	1 (5%)
Mandibular defect class	II	15 (75%)
	IV	5 (25%)
Number of segments	1	1 (5%)
	2	11 (55%)
	3	7 (35%)
	4	1 (5%)

OSCC, oral squamous cell carcinoma; ORN, osteoradionecrosis; MRONJ, medication-related osteonecrosis of the jaw.

The overall median estimated resin volume and printing duration per case were 43.6 ml (13.8–77.4) and 180 minutes (120–255). Median costs per case were EUR 14.30 (4.50–25.30). The median overall operation duration was 650 minutes (480–840) and ischemic time was 165 minutes (90–240). In the class II mandibular defect constellation the operation duration was 630 minutes (480–840) and ischemic time was 150 minutes (90–240). In the class IV mandibular defect constellation the operation duration was 660 minutes (600–750) and ischemic time was 180 minutes (120–185) (*p* = 0.180; *p* = 0.928, respectively).

### The Intraclass Correlation Coefficients

The ICC coefficients (κ) for the horizontal distances head–head medial and lateral showed very good intra- and interrater reliabilities (κ>0.9). The condylar angle measurement showed only satisfactory intra- [between *ICC κ* 0.552, 95% CI -0.292–0.844 and *ICC κ* 0.866, 95% CI 0.655–0.948)] and good interrater reliability (between *ICC κ* 0.845, 95% CI 0.579–0.942), especially in the preoperative measurements. The postoperative condylar angle measurements again showed good to very good agreement (ICC κ>0.9).

ICC coefficients of all three-dimensional parameters (RMSE, MSD, and HD) consistently showed very good intra- and interrater reliability (ICC κ>0.9) (**Supplementary Tables 1** and **2**).

### Conventional and Three-Dimensional Analyses

The detailed results of the conventional pre- and postoperative measurements (horizontal medial and lateral head–head distances and left and right condylar angles) are summarized in **Table 2**.

**Table 2:** Results of conventional and three-dimensional analyses.

Parameter	Absolut median (range)	Median (range) differences	p-value
Pre head–head med	84.4 (76.3–94.4)	-1.9 (-13–8)	0.131^#^
Post head–head med	85.8 (76.0–100.3)		
Pre head–head lat	119.7 (105.3–130.1)	-0.4 (-7.9–6.1)	0.183^#^
Post head–head lat	121.2 (107.2–131.5)		
Pre condyle angle right	23.0 (11.0–37.0)	0.0 (-26–23)	0.142^#^
Post condyle angle right	23.0 (11.3–43.0)		
Pre condyle angle left	-22.0 [-32.0–(-7.0)]	1.4 (22–34.5)	0.042^#^
Post condyle angle left	-20.3 [-56.9–(-8.0)]		
*Parameter*	Median (range)	Parameter	Median (range)
Pre-virt RMSE	1.4 (0.4–7.2)	Pre-post RMSE	2.2 (1.5–11.1)
Pre-virt MSD	0.3 (-0.1–2.9)	Pre-post MSD	0.5 (-0.6–6.1)
Pre-virt HD	0.7 (0.1–3.1)	Pre-post HD	1.5 (1.1–6.5)
Virt-post RMSE	2.3 (1.3–10.7)	Diff RMSE pre-post *vs*. pre-virt	0.7 (-2.7–10.5)
Virt-post MSD	-0.1 (-1.0–5.6)	Diff MSD pre-post *vs*. pre-virt	-0.1 (-1.3–6.0)
Virt-post HD	1.7 (0.1–5.9)	Diff HD pre-post *vs*. pre-virt	0.9 (-0.8–6.4)

Head–head med/lat, medial/lateral horizontal distance between condylar heads; pre-virt, preoperative vs. virtual model; virt-post = virtual vs. postoperative model; pre-post = pre- vs. postoperative model; RMSE, root mean square error; MSD, mean surface distance; HD, Hausdorff distance; Diff, difference.

**^#^** Wilcoxon signed-rank test.

The three-dimensional alignment analyses of the preoperative and virtually planned neomandibula models (= pre-virt = expected deviation from “*ground truth*” model) were associated with a median RMSE of 1.4 (0.4–7.2), MSD of 0.3 (-0.1–2.9), and HD of 0.7 (0.1–3.1). The three-dimensional alignment analyses of preoperative and postoperative models (= pre-post = postoperative, real deviation from “*ground truth*” model) showed a median RMSE of 2.2 (1.5–11.1), MSD of 0.5 (-0.6–6.1), and HD of 1.5 (1.1–6.5) and the differences were significantly different for RMSE (*p* < 0.001) and HD (*p* < 0.001). The difference was not significantly different for MSD (*p* = 0.554).

Three-dimensional alignment analyses of the virtual and postoperative models (= virt-post = postoperative, real deviation from planned situation) had a median RMSE of 2.3 (1.3–10.7), MSD of -0.1 (-1.0–5.6), and HD of 1.7 (0.1–5.9) (**Table 2** and **Figure 3**).

**Figure 4:**
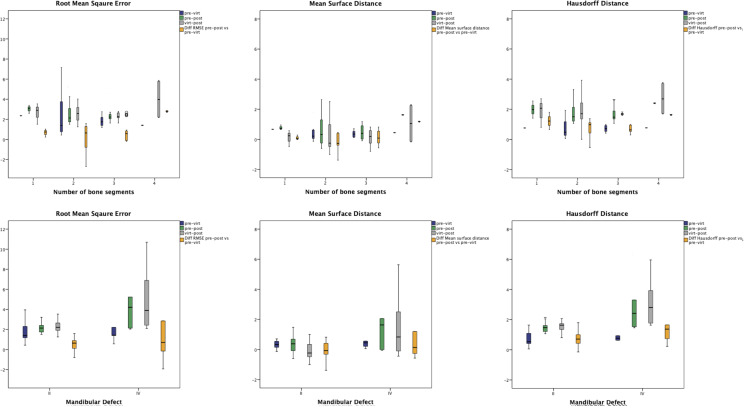
The results for the RMSE, MSD, and HD analyses as a function of number of bone segments (upper row) or mandibular defect class (lower row).

The results for the RMSE, MSD, and HD analyses as a function of number of bone segments or mandibular defect class (II *vs*. IV) are shown in **Figure 4**.

### Uni- and Multivariate Linear Regression Analyses

Uni- and multivariate regression analyses were performed to analyze possible confounding factors (gender, age, indication for surgery, dignity, mandibular defect class, and number of segments) on the RMSE, MSD, and HD of the virtual postoperative model alignment. Gender and number of segments did not have a significant influence on RMSE, MSD, or HD and were thus excluded from the multivariate linear regression analyses. Dignity (benign *vs*. malign) showed a significant influence on RMSE and MSD (*p* = 0.047 95% CI = 0.015–1.987 and *p* = 0.001 95% CI = 0.461–1.767), but not on HD (*p* = 0.223 95% CI = -0.210–0.886). The significant influence of the factors age, indication, dignity, and mandibular defect class remained in the multivariate linear regression analyses (**Table 3**).

**Table 3:** Uni- and multivariable linear regression model of the virtual-postoperative RMSE, MSD, and HD results and possible confounding factors.

Factor	Root mean square error		Mean surface distance		Hausdorff distance	
	*p*-value	95% CI	*p*-value	95% CI	*p*-value	95% CI
**Univariable linear regression model**						
Gender	0.173	-1.561–0.286	0.056	-1.246–0.017	0.148	-0.874–0.135
Age	<0.001	-0.094–(-0.046)	<0.001	-0.054–(-0.018)	<0.001	-0.051–(-0.024)
Indication	<0.001	0.253–0.696	0.026	0.023–0.353	<0.001	0.165–0.402
Dignity	0.047	0.015–1.987	0.001	0.461–1.767	0.223	-0.210–0.886
Mandibular defect type	<0.001	1.092–1.916	<0.001	0.568–1.192	<0.001	0.638–1.075
Number of bone segments	0.416	-1.008–0.421	0.908	-0.526–0.468	0.577	-0.502–0.282
**Multivariable linear regression model**						
Age	0.01	-0.055–(-0.016)	0.109	-0.029–0.003	0.001	-0.029–(-0.007)
Indication	0.004	0.123–0.602	0.022	0.034–0.426	0.006	0.057–0.331
Dignity	<0.001	1.076–2.654	<0.001	1.056–2.347	/	/
Mandibular defect class	<0.001	0.597–1.391	<0.001	0.298–0.947	<0.001	0.342–0.796

## Discussion

This study is one of the few studies on in-house CAD/CAM solutions that presents a critical contemporary three-dimensional evaluation of virtually planned mandibular reconstructions with FFF. Tarsitano et al. described that the use of CAD/CAM-assisted mandibular reconstruction is economically viable, as the money saved by the reduction of operation duration offsets the associated costs of approximately EUR 3,450 (5). Rommel et al. calculated the increased costs for planning and the use of patient-specific cutting guides offered and manufactured by osteosynthesis manufacturers to be EUR 2,250 per case (3). According to reviews and meta-analyses others reported the cost of virtual surgical planning to range from USD 3,000–8,200 (6, 10). These higher costs can be explained by the fact that the studies included in the reviews/meta-analyses were published between 2013 and 2016, at a time when 3D printing, for example, was even more expensive than it is today, and usually included the production of patient-specific reconstruction plates. Costs resulting from cost-effective in-house design and printing solutions are reported to be significantly lower. Bosc et al. calculated their cost per case for in-house design and printing at EUR 989 (13). In contrast, our reported median costs per case were EUR 14.30 (4.50–25.30). This represents an excellent cost–benefit ratio, We have deliberately omitted recent non-negligible cost items (acquisition and maintenance costs, sterilization time, and planning time of approximately five to six hours, salary, etc.) and did also not calculated overall potential cost savings for simplicity, as have many other authors, and focused exclusively on material costs. More recently, Moe et al. reported their cost per case to be USD 3.87 for in-house design and printing. Dell’Aversana Orabona et al. reported a cost per case of EUR 3 (27). At EUR 14.30, the calculated costs per case are comparable with data from the literature and are completely negligible in relation to the surgical costs. Our slightly higher costs can be explained by two factors: we created and printed repositioning aids for each osteotomy site, and the neomandibula segment was also printed in order to bend the miniplates preoperatively.

The total lead time (planning, designing, printing, post-processing, final preparation with drill sleeves, and sterilization) is two to three days, which agrees very well with the times reported in the literature (13, 14) – and can be expedited further with growing experience and optimization of the processes. Another essential aspect raised by Numajiri et al. is that this form of cost-effective planning is done in the surgeon’s time and is not outsourced to the osteosynthesis manufacturers and their clinical engineers as is usually the case (28). Geusens et al. reported that they have employed a full-time clinical engineer, which could be another solution to the altruistic behavior of interested and motivated surgeons (21). According to Tang et al., the economic benefits and limitations associated with the application of virtual surgical planning must be weighed against patient outcomes (4). The introduction of in-house solutions may represent the ideal approach in this respect, as costs can be saved and treatment optimized.

The study design and methodical analyses of the mandibular reconstruction is generally heterogenous among the available studies (4), which makes a direct comparison difficult. In a recent systematic review and meta-analysis on virtual surgical planning in mandibular reconstruction Barr et al. were not able to define a valuable parameter and result for “*accuracy*”, even though many authors highlight the introduction of CAD/CAM algorithms as beneficial (6). The reason is again the heterogeneity of parameters. The majority of studies compared the preoperative virtual plan to the postoperative situation by measuring the fibula segment lengths (21, 29–31), point-to-point distances and angles (3, 13, 14), intercondylar distance (32), or intercoronoid distance (21) or by comparing interfragmentary gap distances (33). Numajiri et al. analyzed an algorithm for the production of low-cost cutting guides designed and printed in-house (28). They reported an error deviation of 2.4 mm in 12 studied *in-vitro* model surgeries. For this laboratory setting under ideal conditions, an error deviation of 2.4 mm seems to be quite high. Four years later, Numajiri et al. published a clinical study comparing freehand and in-house-planned CAD/CAM FFF cases (14). Based on a point-to-point analysis, they describe their in-house Blender-based solution as accurate. Interestingly, the results of the more recent study are more precise than the results of their *in vitro* studies, which can probably be attributed to the research group’s growing experience with CAD/CAM-based surgical planning and the improvement of the processes. Yu et al. aligned the virtual plan and the postoperative result. But the evaluation was again limited only to distance measurements between corresponding points (8). Bosc et al. analyzed their postoperative results of in-house (Meshmixer or Blender) planned and printed cutting guides after surface alignment using CloudCompare, another potent open-source software solution for this purpose (13). However, the authors only used surface matching to get the maximum points of convergence for better correspondence of analyzed distances and angles. In all studies mentioned, the deviations between planning and operative results were 1–3 mm.

The disadvantage of all “conventional” measurement methods, which essentially involve only the analysis of distances (point-to-point or bone segment lengths) and angles, is that the effect of a small deviation on the entire three-dimensional geometry is neglected. An involuntary deviation from the planned osteotomy angle, fibula segment length, or fibula segment position will result in the derotation of the remaining mandible, and consequently in changes to the condyle angle.

For this reason and unlike most studies that have performed analyses of CAD/CAM-assisted mandibular reconstructions with the FFF, our focus has been on a three-dimensional analysis that encompasses the entire mandibular geometry. For example, Wallner et al. and van Eijnatten et al. applied three-dimensional parameters to compare the accuracy and comparability of open-source software solutions and influence of threshold setting for DICOM data set segmentation (23, 24). This approach seems to be a contemporary and objective way to compare two similar and rather complex objects but has not yet been used routinely in analyses of mandibular reconstructions (11, 12, 27). Each of our examined three-dimensional parameters – RMSE, MSD, and HD – had both excellent intra- and interobserver reliability, making the measured results valid for further analyses and discussion. In contrast, the conventional measurement of condylar angles in particular showed poorer intraobserver reliability for both examiners, which was confirmed by comparable interobserver reliability (**Supplementary Tables 1**, **2**). Horizontal distances also revealed very good intra- and interobserver reliabilities. Dell’Aversana Orabona et al. used the open-source software InVesalius for in-house planning in four consecutive cases (27). They reported a distance between three-dimensional pre- and postoperative mesh points of 1.63 mm and a standard deviation of 5.45 mm in a volume overlay analysis. Recently, Moe et al. published results about their in-house workflow and described a mean surface overlay difference of 1.90 mm with an RMSE of 3.72 mm for 29 virtually planned cases, including 24 FFF (12). These results coincide with ours (virt-post MSD -0.1 (-1.0–5.6); virt-post RMSE 2.3 (1.3–10.7)). A novel aspect of our study is the comparison of preoperative situation and virtual model (pre-virt), which reflects the expected deviation from the “ground truth” model. Consequently, the difference between pre-post and pre-virt also reflects the accuracy when the value reaches 0. For this new parameter, we revealed excellent results for RMSE, MSD, and HD (**Table 2**).

In addition to potential cost savings and increased accuracy, we believe that this in-house planning has a great advantage especially in the training of junior surgeons. Dealing with virtual planning leads to the trainees intensively dealing with the clinical case, and potentially developing a higher demand for the final surgical result. Therefore, in our department, junior surgeons are introduced to virtual in-house fibula planning and learn to independently plan and create resection and repositioning guides. Unlike clinical engineers, they also perform flap elevation and mandibular reconstruction under the guidance of experienced specialists, so they will be involved in all steps of these complex reconstruction cases at an early stage in their careers. But this aspect (e.g. evaluation of junior surgeon´s satisfaction, confidence or virtual planning) was no not evaluated this study.

### Limitations, Pitfalls and Solutions

A major drawback of this study was the application of an license-based segmentation software that is associated with an additional acquisition cost (Mimics^®^ 17.0, Materialise; Leuven, Belgium with an one-time acquisition cost in 2014 of EUR 11,700). But, we used Mimics in order to reduce potential software-based pitfalls in our in-house workflow. Nevertheless, there exist reliable and accurate open-source solutions for segmentation like Slicer, as described by Wallner et al. and Egger et al. (23, 34, 35).

**Figure 5:**
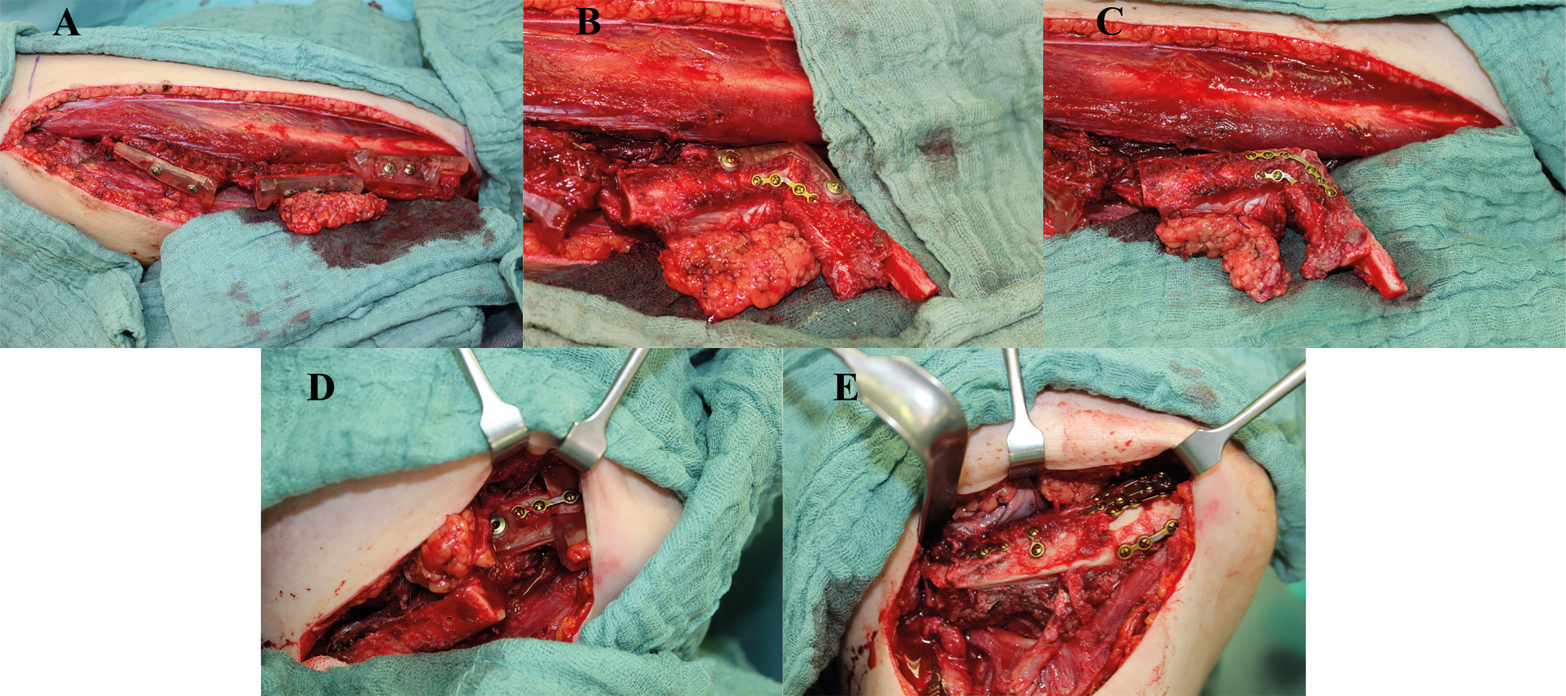
Clinical example with intraoperative images of a case with hemimandibulectomy with exarticulation (Brown IIc) due to chronic osteomyelitis. Reconstruction was performed with a three-segment free fibular flap (FFF), with the corpus section reconstructed with a double-barrel component for better neomandibula contouring and crestal bone position for secondary enossal implant insertion. **(A)** FFF following osteotomies with temporarily applied cutting guides, **(B, C)** reconstruction of the ramus including neo-condylar head and crestal corpus section applying a repositioning aid and pre-bent 2.0 miniplate osteosynthesis, **(D)** positioning of the neomandibula with another repositioning aid, and **(E)** final reconstructive result with the double-barreled caudal FFF section for optimized bony countering.

Through precise planning the original position of the mandible should be imitated as closely as possible from an esthetic and functional point of view. With regard to occlusion and jaw movement, the correct position of the temporomandibular joint is important, and the course of the neo-alveolar ridge is decisive for subsequent dentoalveolar rehabilitation, while from an esthetic point of view the projection of the chin and jaw angle region is particularly decisive. After a learning period, the planning of complex and heterogenous reconstructive cases with the open-source software Blender was feasible and led to excellent results with regard to the realization of virtual plans (**Figure 5**). But incorrect virtual planning can lead to poor postoperative results even if all surgical steps are basically correct and performed as planned. In the experience of the authors, one key pitfall region is the mandibular ramus. When reconstructing it, the transition of the outer surfaces of the fibula and the remaining cranial ramus portion must blend perfectly. If this is not the case, the application of the osteosynthesis will derotate the ramus portion and consequently change the condyle angle and position. But condylar position changes may occur also later in a longer observational interval than it was in our study (36). But nevertheless, a derotation of the condylar head because of the cranial ramus rotation will be visible immediately. The functional sequalae is uncertain and needs further evaluation (37). A further prerequisite is the optimal design of ramus/collum resection guides. Aspects that should be taken into account here are that the mandibular angle is gripped caudally, so that the correct vertical positioning of the guide is ensured. In addition, the guide must not be too thick cranially to allow sawing; if necessary, an angled micro saw can be used, which is usually not a problem with regard to the general bone thickness in this area.

Lastly, we think that the usage of repositioning guides is an excellent alternative to intraoperative navigation as described by Yu et al. (8) to critically feedback the reconstructive result intraoperatively. Especially the combination of repositioning guides and pre-bent osteosynthesis plates enhance the overall accuracy. This is also reflected by the fact that the number of bone segments had no significant effect on RMSE, MSD, or HD (**Table 3**) and might explain our better results for MSD and RMSE compared to Dell’Aversana Orabona et al. and Moe et al. (12, 27). Regarding the design of the repositioning guides, we recognized that we achieved better fit of the guides when designing multiple, osteotomy-specific guides rather than one large, contiguous guide. The supposed inaccuracy of the large repositioning guides is most likely due to 3D printing itself.

## Conclusion

After a certain learning period, open-source software facilitates cost-effective and precise in-house virtual planning of mandibular reconstructions with a short lead time and without the need for external companies. Even highly complex reconstructions are thus possible with favorable results. In addition, the open-source software offers an excellent possibility to illustrate the surgical procedure. This might enhance the understanding for younger colleagues and increase their likelihood and frequency of an ideal surgical result.

## Data Availability Statement

The raw data supporting the conclusions of this article will be made available by the authors, without undue reservation.

## Ethics Statement

The studies involving human participants were reviewed and approved by The institutional ethics committee of the Technical University of Munich, Klinikum rechts der Isar (Approval number: 326/21 S-EB). Written informed consent for participation was not required for this study in accordance with the national legislation and the institutional requirements.

## Author Contributions

LR: Study design/conduction, operations, data interpretation, and major contribution to manuscript writing and revision. PK: Surface matching and analysis, data acquisition and interpretation, contribution to revision. FG: Surface matching and analysis, data acquisition and interpretation, statistical analysis and contribution to revision. MS: Creation of figures, statistical analysis, contribution to revision. JW: Study design, data interpretation, contribution to revision. MN: Data interpretation, contribution to revision. K-DW: Data interpretation, contribution to revision. AF: Study design/conduction, operations, data interpretation, and major contribution to manuscript writing. All authors contributed to the article and approved the submitted version.

## Conflict of Interest

The authors declare that the research was conducted in the absence of any commercial or financial relationships that could be construed as a potential conflict of interest.

## Publisher’s Note

All claims expressed in this article are solely those of the authors and do not necessarily represent those of their affiliated organizations, or those of the publisher, the editors and the reviewers. Any product that may be evaluated in this article, or claim that may be made by its manufacturer, is not guaranteed or endorsed by the publisher.
