# Supplementary material for: In-House, Open-Source 3D-Software-Based, CAD/CAM-Planned Mandibular Reconstructions in 20 Consecutive Free Fibula Flap Cases: An Explorative Cross-Sectional Study With Three-Dimensional Performance Analysis
Source: Front Oncol. 2021 Sep 24;11:731336. doi: 10.3389/fonc.2021.731336 (PMC8498593; doi:10.3389/fonc.2021.731336)
Supplement: Supplementary file 1 [file DataSheet_1.pdf]

Supplementary Table legends

Supplement Table 1. Intraclass correlation (ICC) coefficient (Cohen's kappa = κ) to analyze the *intrarater* reliability of measurements performed by the two independent raters (PK and FDG) applying a two-way mixed model.

Supplement Table 2. Intraclass correlation (ICC) coefficient (Cohen's kappa = κ) to analyze the *interrater* reliability of measurements performed by the two independent raters (PK and FDG) applying a two-way mixed model.

List of Supplementary Tables

**Supplementary Table 1.** Intraclass correlation (ICC) coefficient (Cohen's kappa = κ) to analyze the *intra-rater* reliability of measurements performed by the two independent raters (PK and FDG) applying a two-way mixed model.		
Parameter	***Rater 1***	***Rater 2***
	ICC κ (95% CI)	***ICC κ (95% CI)***
**Pre head–head med**	0.999 (0.990–0.999)	0.988 (0.966–0.995)
**Pre head–head lat**	0.999 (0.996–0.999)	0.994 (0.986–0.998)
**Pre condyle angle right**	0.747 (0.330–0.904)	0.552 (-0.292–0.844)
**Pre condyle angle left**	0.866 (0.655–0.948)	0.693 (0.078–0.897)
**Post cap–cap med**	0.997 (0.985–0.999)	0.963 (0.905–0.986)
**Post cap–cap lat**	0.997 (0.992–0.999)	0.998 (0.995–0.999)
**Post condyle angle right**	0.861 (0.612–0.951)	0.942 (0.840–0.979)
**Post condyle angle left**	0.951 (0.839–0.983)	0.945 (0.848–0.980)
**Pre-virt RMSE**	0.958 (0.891–0.984)	0.995 (0.988–0.998)
**Pre-virt MSD**	0.977 (0.940–0.991)	0.999 (0.997–0.999)
**Pre-virt HD**	0.937 (0.835–0.976)	0.967 (0.917–0.987)
**Virt-post RMSE**	0.959 (0.892–0.984)	0.948 (0.867–0.979)
**Virt-post MSD**	0.964 (0.906–0.987)	0.932 (0.827–0.973)
**Virt-post HD**	0.930 (0.812–0.974)	0.937 (0.840–0.975)
**Pre-post RMSE**	0.998 (0.995–0.999)	0.989 (0.972–0.995)
**Pre-post MSD**	0.996 (0.989–0.998)	0.995 (0.987–0.998)

**Pre-post HD**	0.998 (0.995–0.999)	0.988 (0.971–0.995)

Abbreviations:

Head–head med/lat = medial/lateral horizontal distance between condylar heads; pre-virt = preoperative vs. virtual model; virt-post = virtual vs. postoperative model; pre-post = pre- vs. postoperative model; RMSE = root mean square error; MSD = mean surface distance; HD = Hausdorff distance

Supplementary Table 2. Intraclass correlation (ICC) coefficient (Cohen's kappa = κ) to analyze the *interrater* reliability of measurements performed by the two independent raters (PK and FDG) applying a two-way mixed model.

Parameter	***ICC κ***	***95% CI***
**Pre head–head med**	0.996	0.990–0.998
**Pre head–head lat**	0.997	0.985–0.999
**Pre condyle angle right**	0.845	0.579–0.942
**Pre condyle angle left**	0.907	0.750–0.965
**Post cap–cap med**	0.993	0.981–0.997
**Post cap–cap lat**	0.999	0.997–1.000
**Post condyle angle right**	0.926	0.763–0.974
**Post condyle angle left**	0.909	0.755–0.967
**Pre-virt RMSE**	0.989	0.973–0.996
**Pre-virt MSD**	0.994	0.985–0.998
**Pre-virt HD**	0.976	0.940–0.991
**Virt-post RMSE**	0.985	0.962–0.994
**Virt-post MSD**	0.999	0.997–1.000
**Virt-post HD**	0.964	0.909–0.986
**Pre-post RMSE**	0.998	0.994–0.999
**Pre-post MSD**	0.995	0.988–0.998
**Pre-post HD**	0.990	0.976–0.996

Abbreviations:

Head–head med/lat = medial/lateral horizontal distance between condylar heads; pre-virt = preoperative vs. virtual model; virt-post = virtual vs. postoperative model; pre-post = pre- vs. postoperative model; RMSE = root mean square error; MSD = mean surface distance; HD = Hausdorff distance
