# Supplementary material for: In-House, Open-Source 3D-Software-Based, CAD/CAM-Planned Mandibular Reconstructions in 20 Consecutive Free Fibula Flap Cases: An Explorative Cross-Sectional Study With Three-Dimensional Performance Analysis
Source: Front Oncol. 2021 Sep 24;11:731336. doi: 10.3389/fonc.2021.731336 (PMC8498593; doi:10.3389/fonc.2021.731336)

Supplementary Data

A step-by-step guide for in-house Blender-based free fibula flap planning

General considerations

Principles – fibula positioning

- Pedicle always lingually
- Facies lateralis always buccally

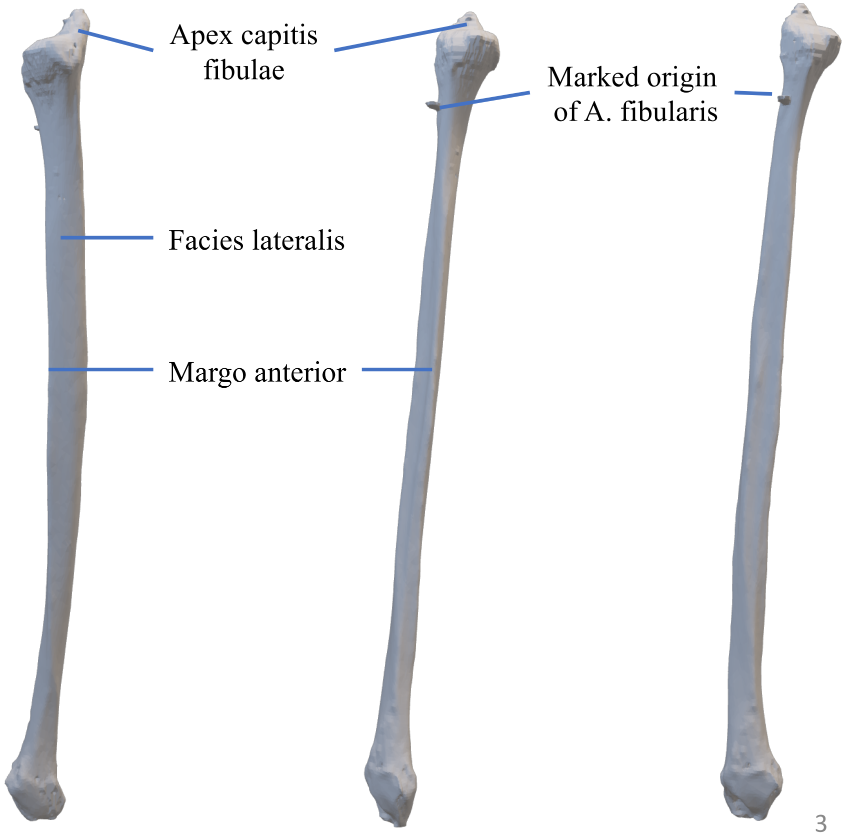

Principles – cutting guides

- *Fibula*
  - Margo anterior as guiding rim
  - “dog bone like” design
  - Drilling holes

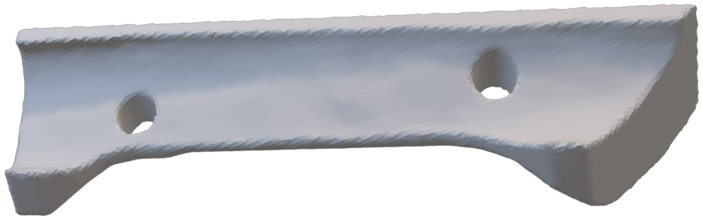

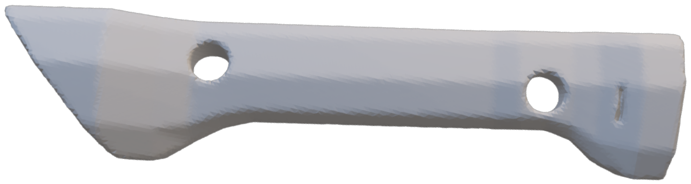

- *Mandible*
  - Extraoral resection: single guides or one block guide
  - Intraoral resection: block guide and slit
  - Drilling holes

Blender – important commands

- *Obejct Mode*
  - Select and deselect
  - “S” - scale
  - Add Modifier → Boolean operation → Intersect/Difference/Union

- *Edit Mode*
  - ctrl+R → subdivide
  - Movements of points, lines or areas

Begin planning

Start – import

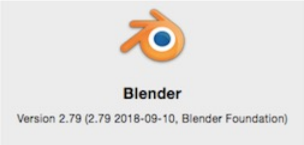

- Import STL file

- Command: Transform – Geometry to Origin

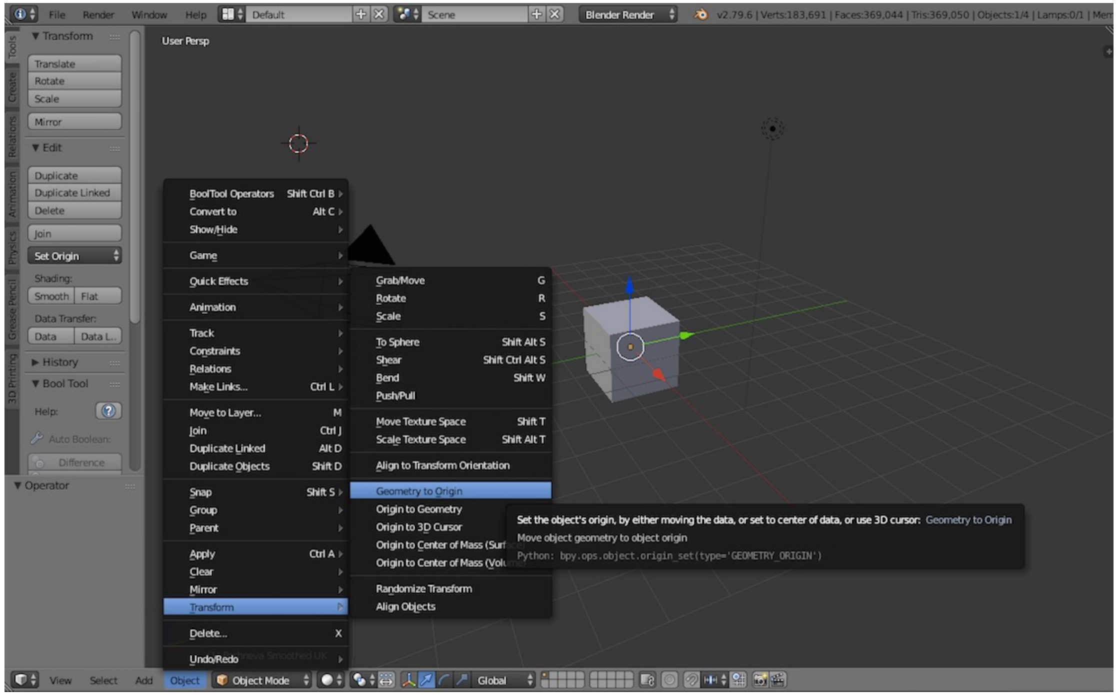

Start – orientation

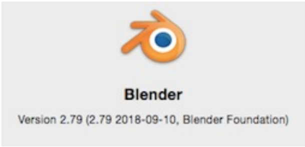

- Model orientation with Numpad numbers 1, 3 and 7

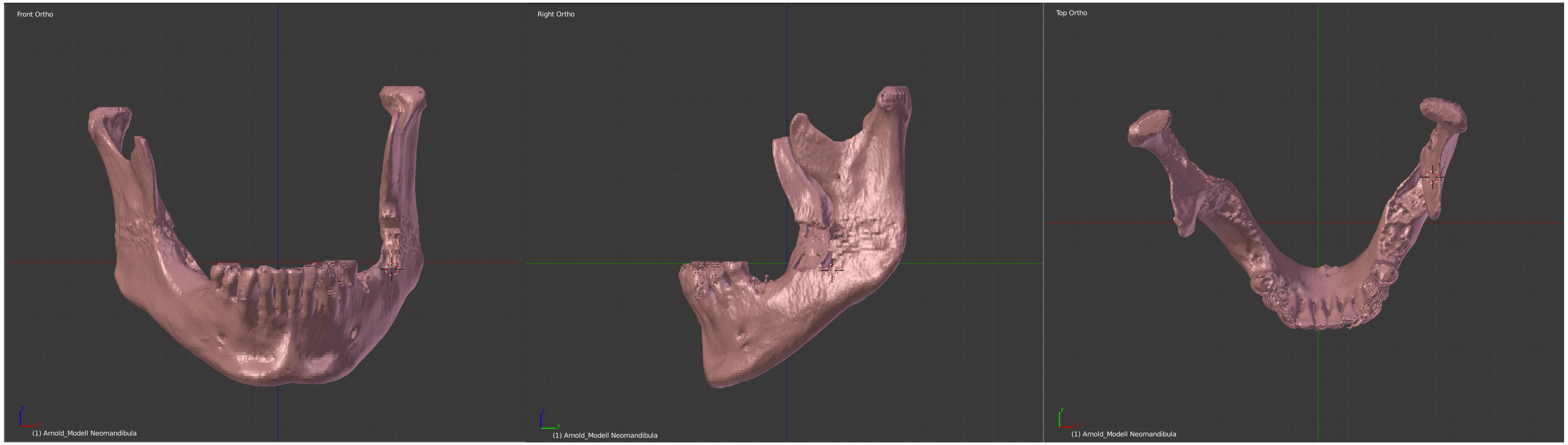

1 **3** **7**

Define cutting planes

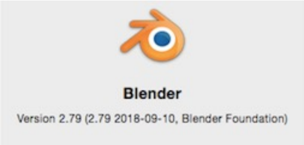

- Shift+A: Add Cube

- Cube´s depth 0.1 mm

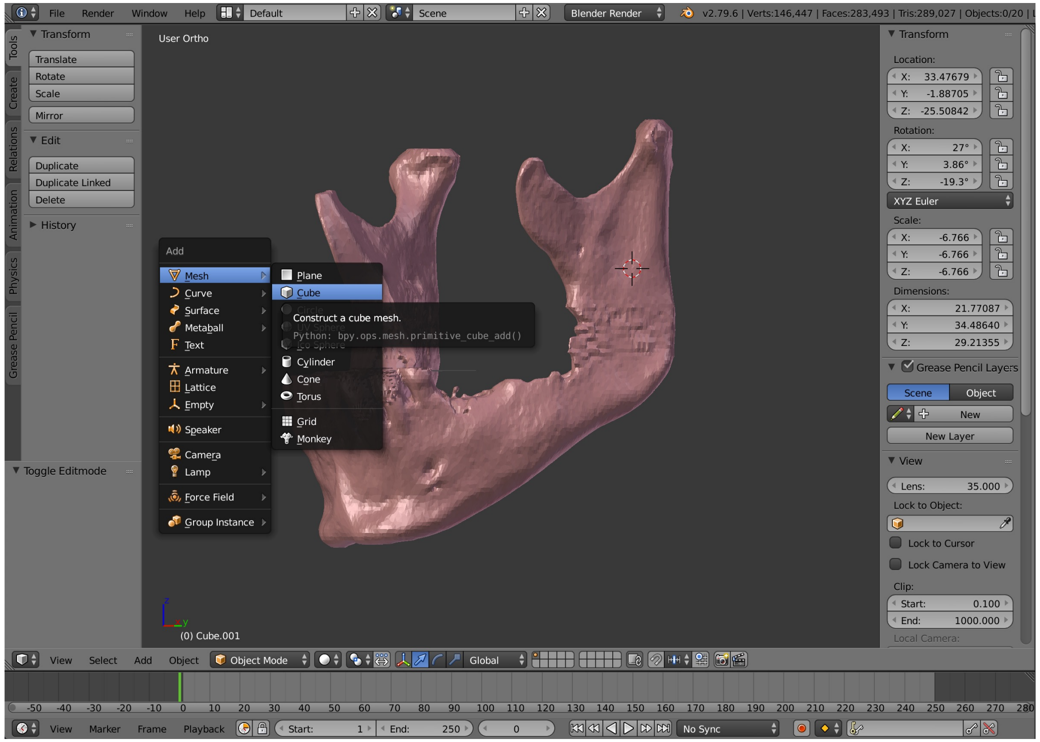

Define cutting planes

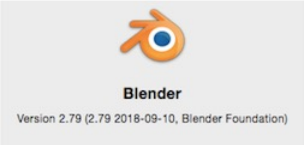

- Shift+A: Add Cube
- Cube´s depth 0.1 mm
- Position cutting plane
- Duplicate cutting plane

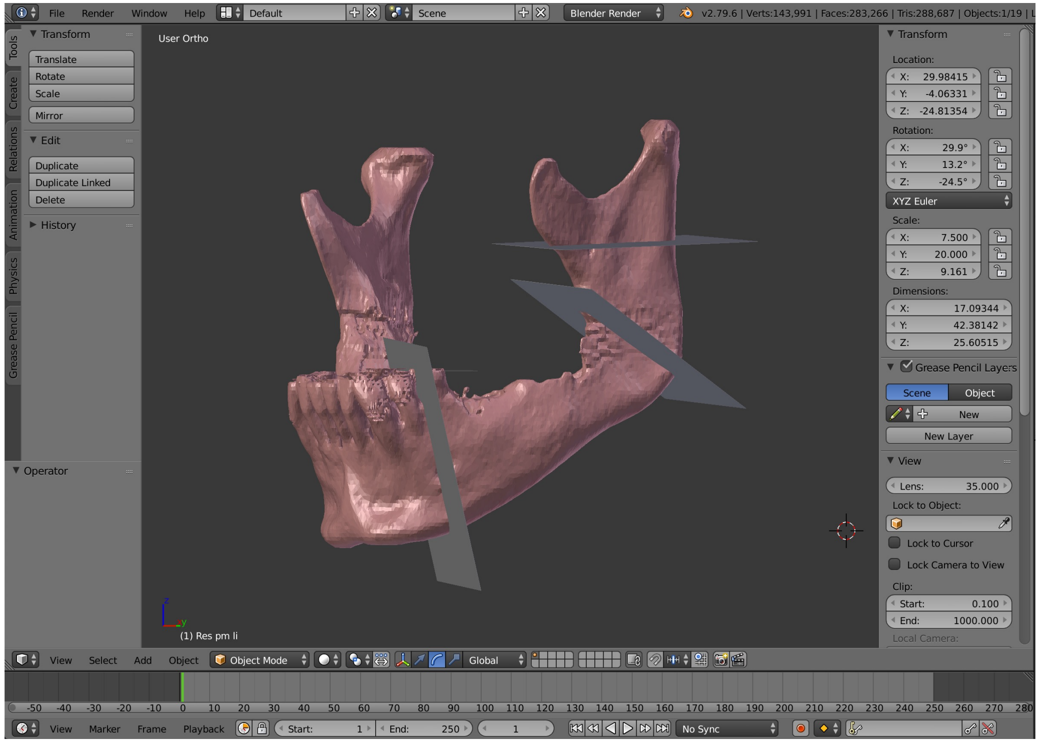

Define fibula positions

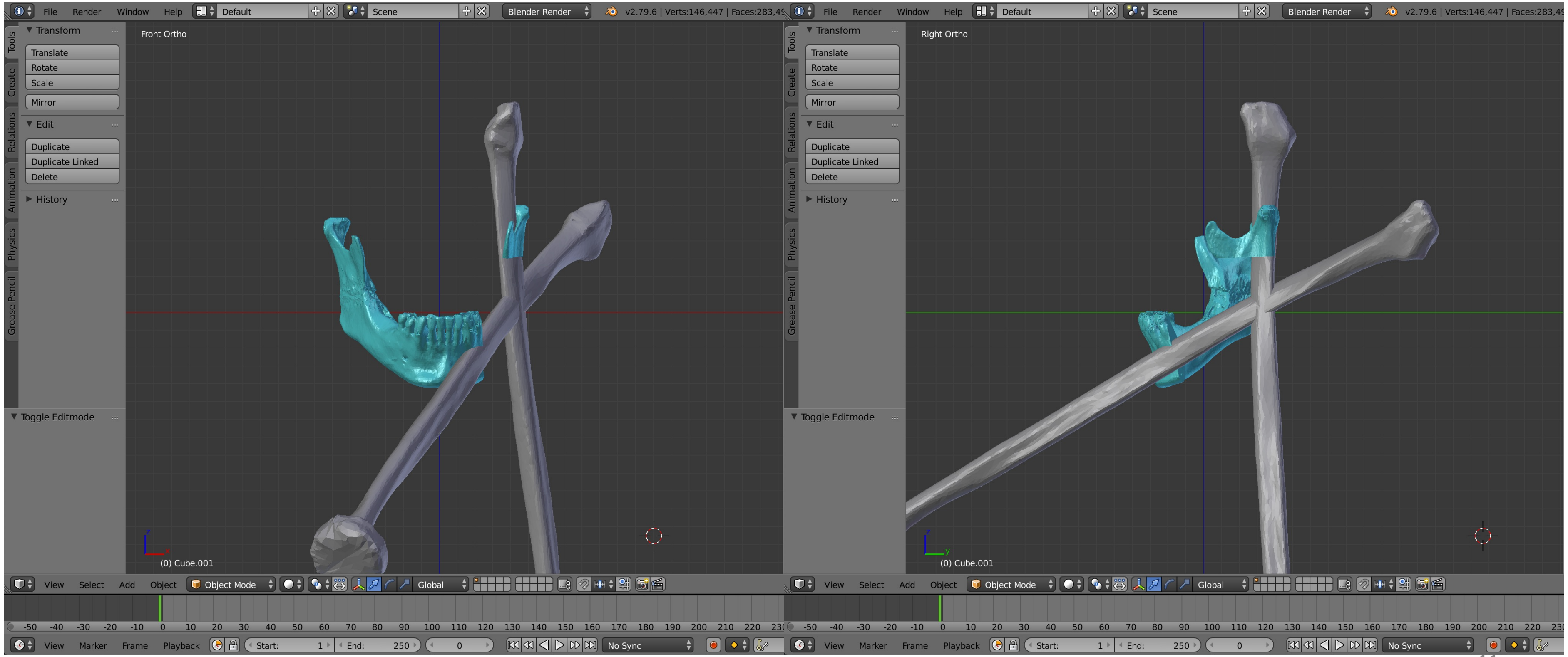

Simulate fibula positions → Neomandibula

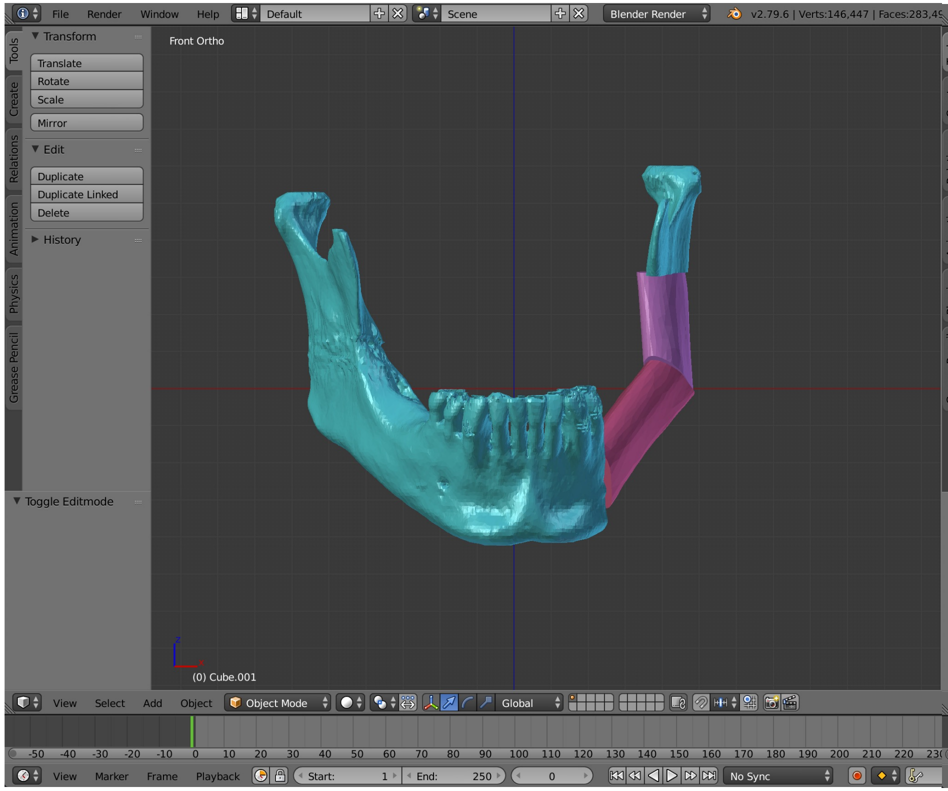

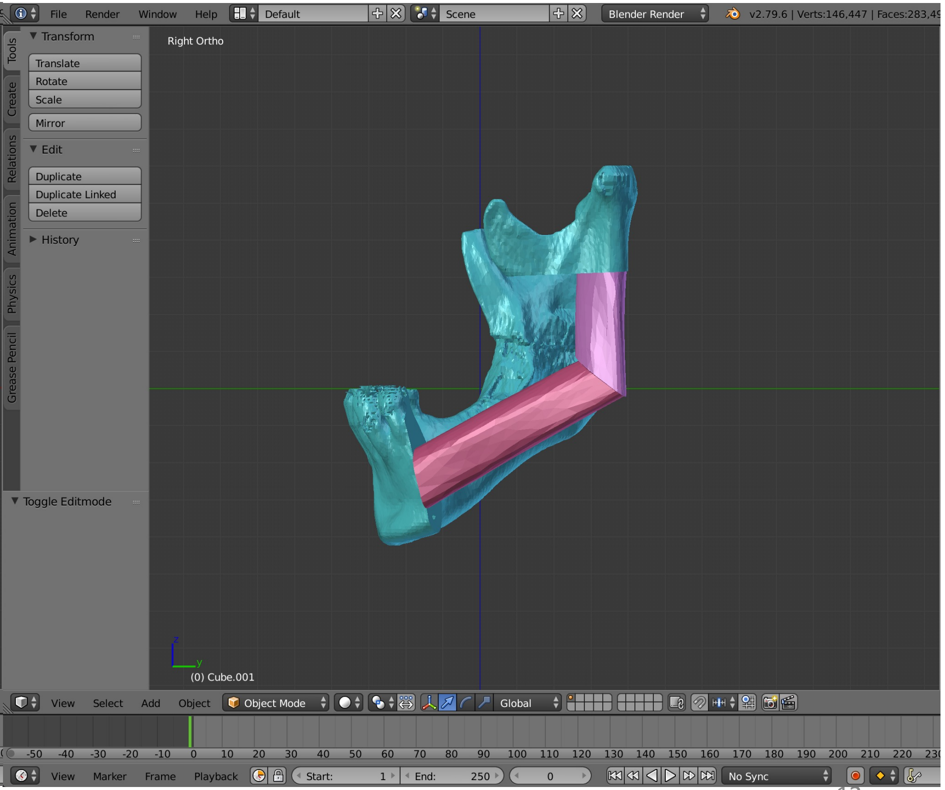

General principles of cutting guide design

Cutting guides – Design

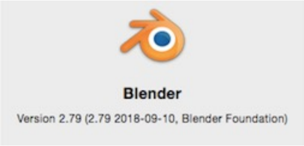

Object Mode — *Shift+A:* add Cube

Object Mode — Cube orientation

Edit Mode — *Ctrl+R:* subdivide

Edit Mode — Modify form manually

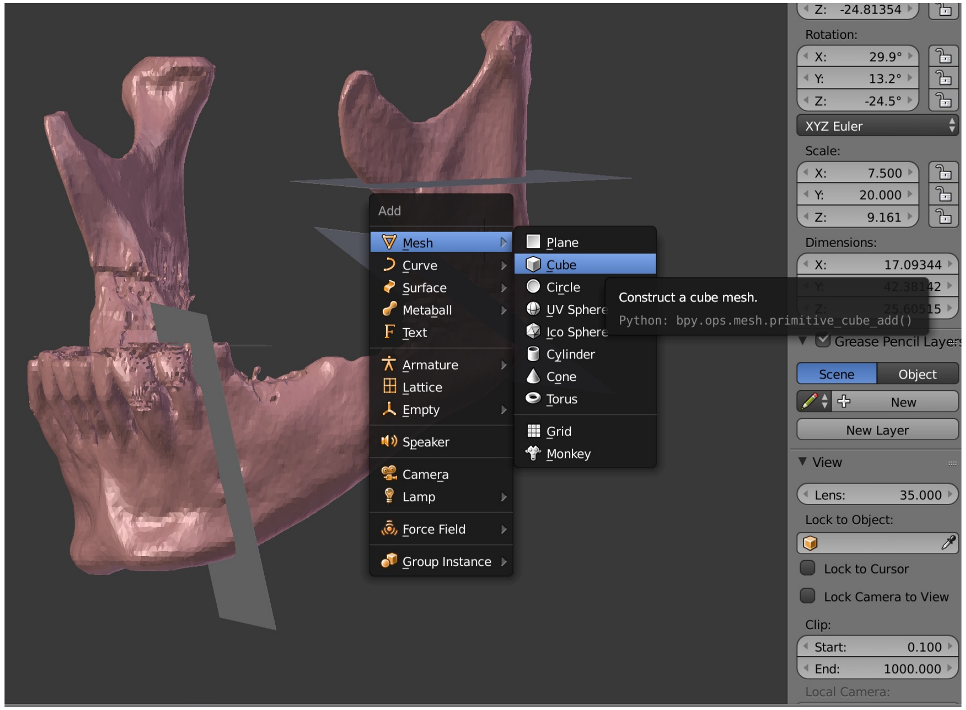

Cutting guides – Design

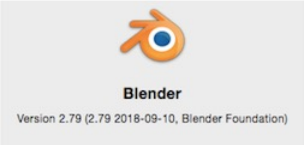

- *Object Mode* → *Shift+A*: add Cube
- *Object Mode* → Cube orientation
- *Edit Mode* → *Ctrl+R*: subdivide
- *Edit Mode* → Modify form manually

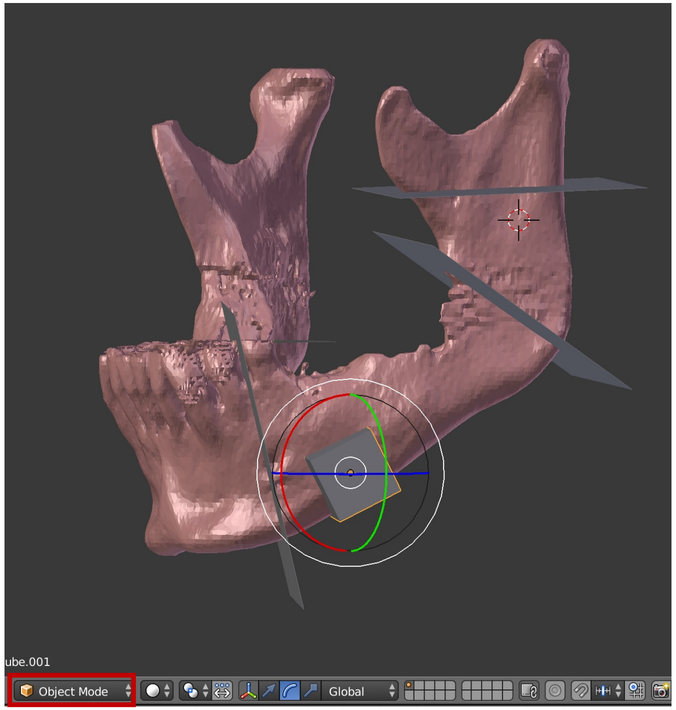

Cutting guides – Design

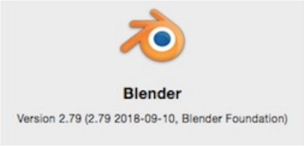

- *Object Mode* — *Shift+A:* add Cube
- *Object Mode* — Cube orientation
- *Edit Mode* — *Ctrl+R:* subdivide
- *Edit Mode* — Modify form manually

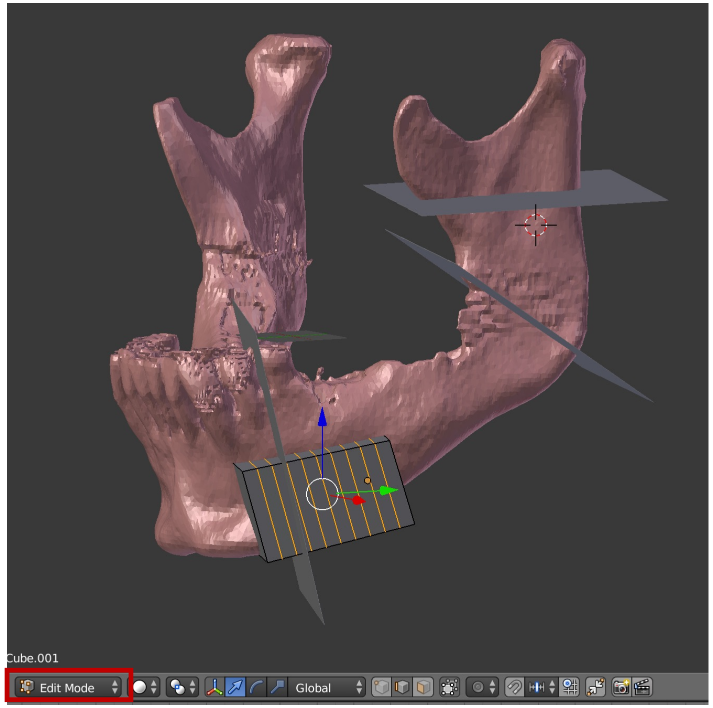

Cutting guides – Design

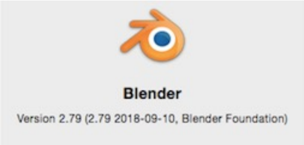

- **Object Mode** → *Shift+A:* add Cube
- **Object Mode** → Cube orientation
- **Edit Mode** → *Ctrl+R:* subdivide
- **Edit Mode** → Modify form manually

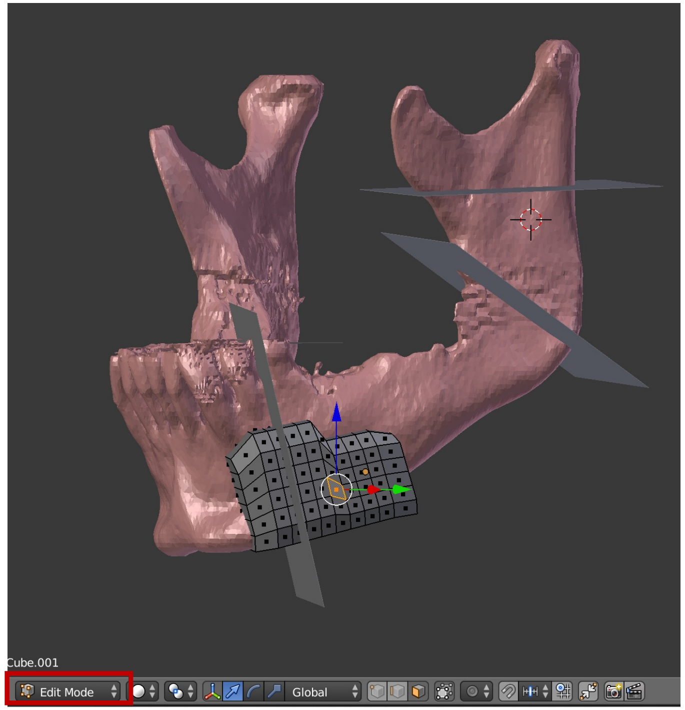

Cutting guides – Boolean in *Object Mode*

- Mark cutting guide → *Add Modifier* → *Boolean*
- *Difference* (guide - mandible) → Apply
- Boolean operations for mandibular and fibular cutting guides

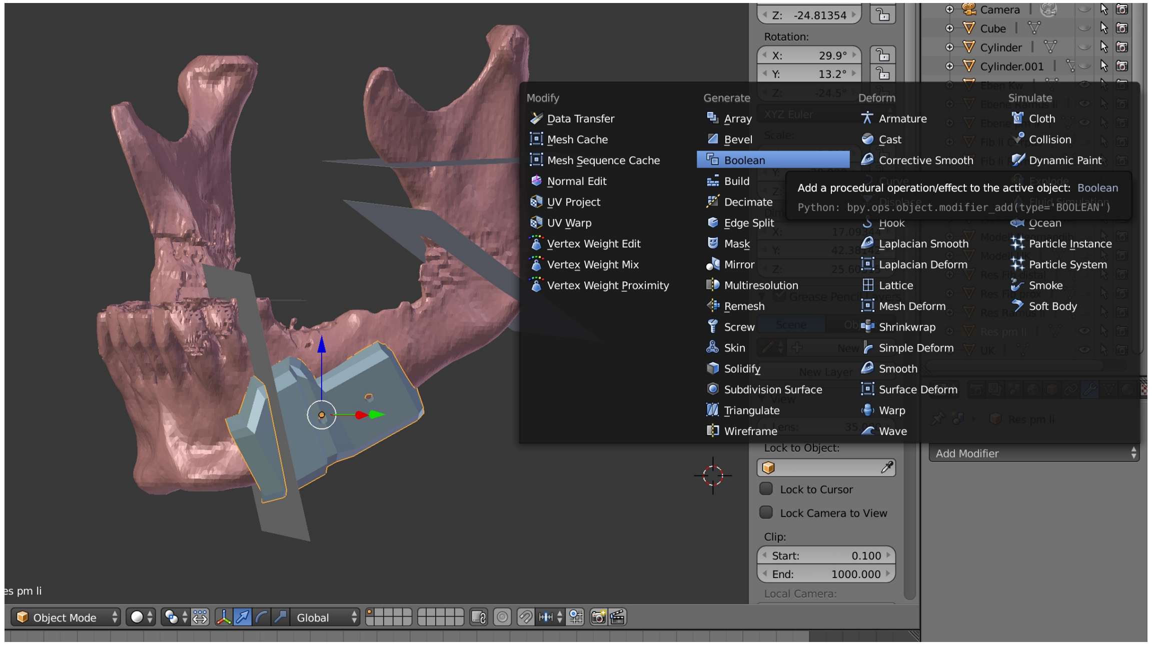

Cutting guides – Boolean in *Object Mode*

- Mark cutting guide → *Add Modifier* → *Boolean*
- *Difference* (guide - mandible) → Apply
- Boolean operations for mandibular and fibular cutting guides

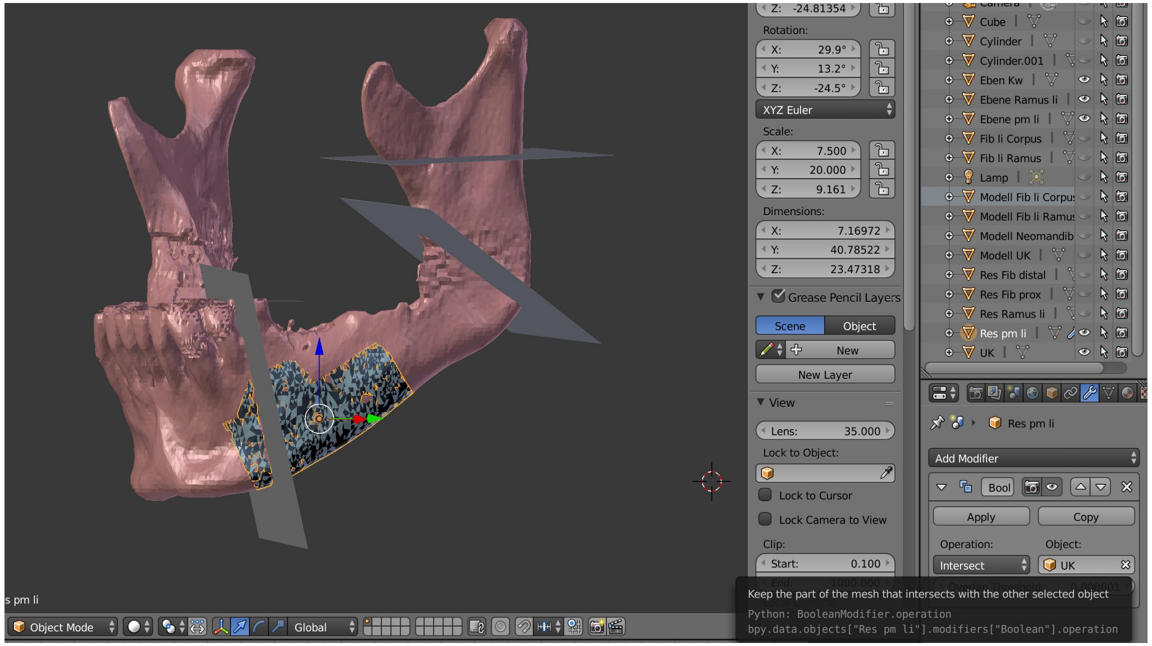

Cutting guides – Boolean in *Object Mode*

- Mark cutting guide → *Add Modifier* → *Boolean*
- *Difference* (guide - mandible) → Apply
- Boolean operations for mandibular and fibular cutting guides

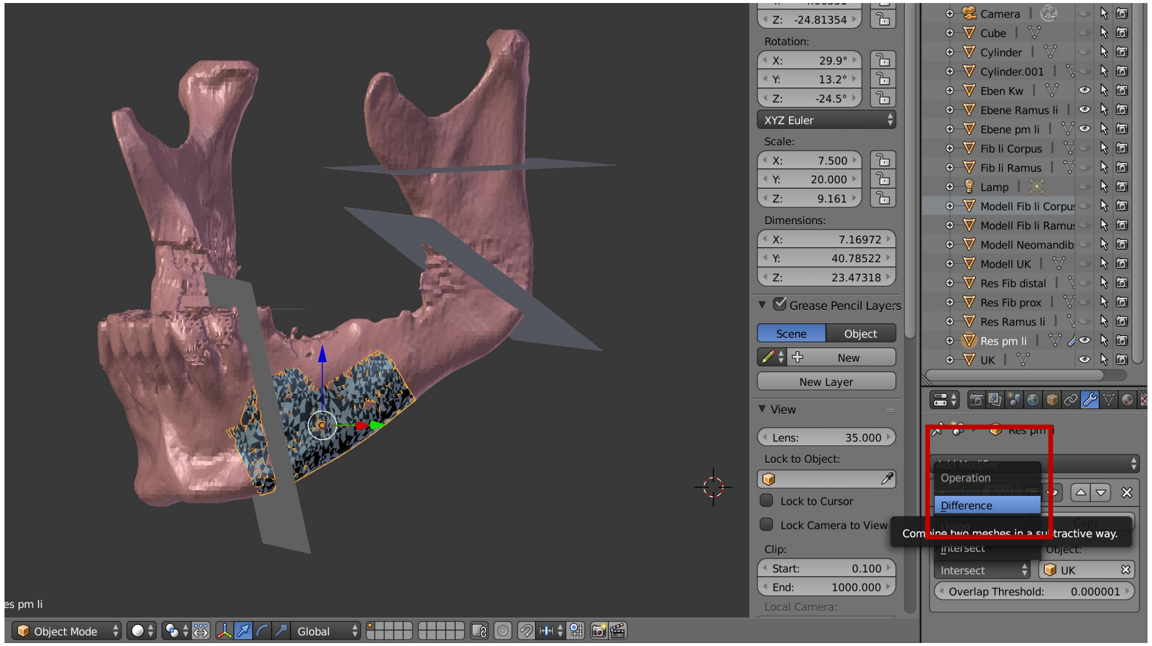

Cutting guides – Boolean in *Object Mode*

- Mark cutting guide → *Add Modifier* → *Boolean*
- *Difference* (guide - mandible) → Apply
- Boolean operations for mandibular and fibular cutting guides

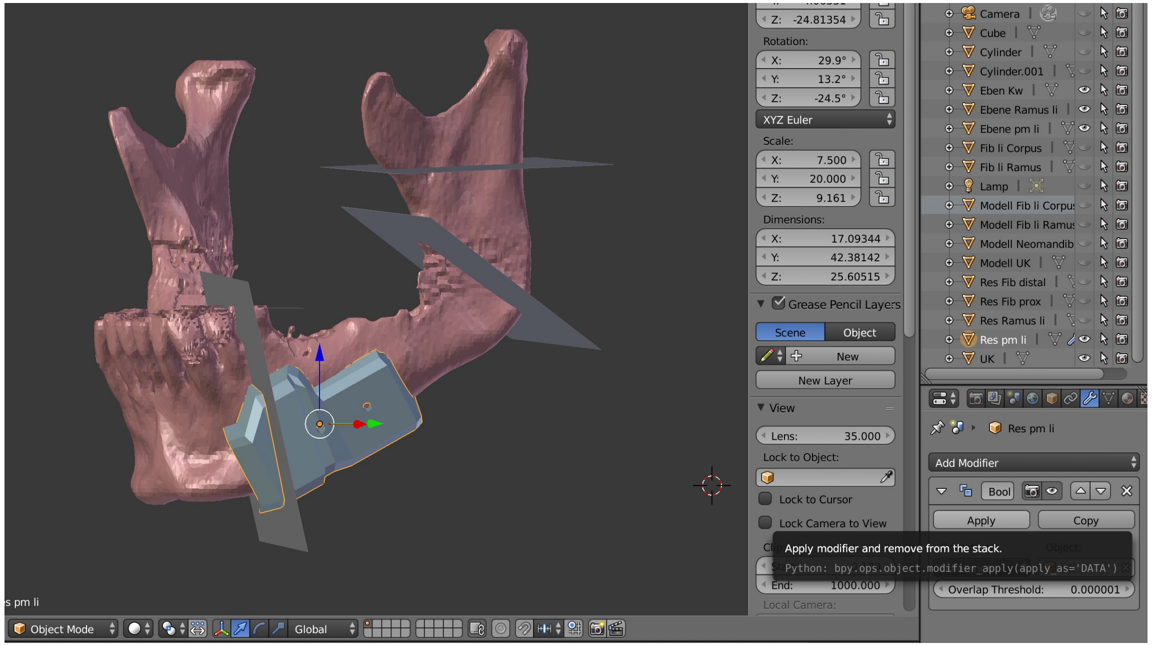

Cutting guides – Labelling

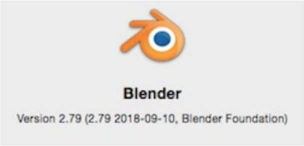

- Shift+A – Text
- *Edit Mode:* Modify text
- Extrude
- Position
- Transform Text into Mesh:
  - Alt+C – *Curves intro Mesh*
- Boolean: Difference guide-text mesh

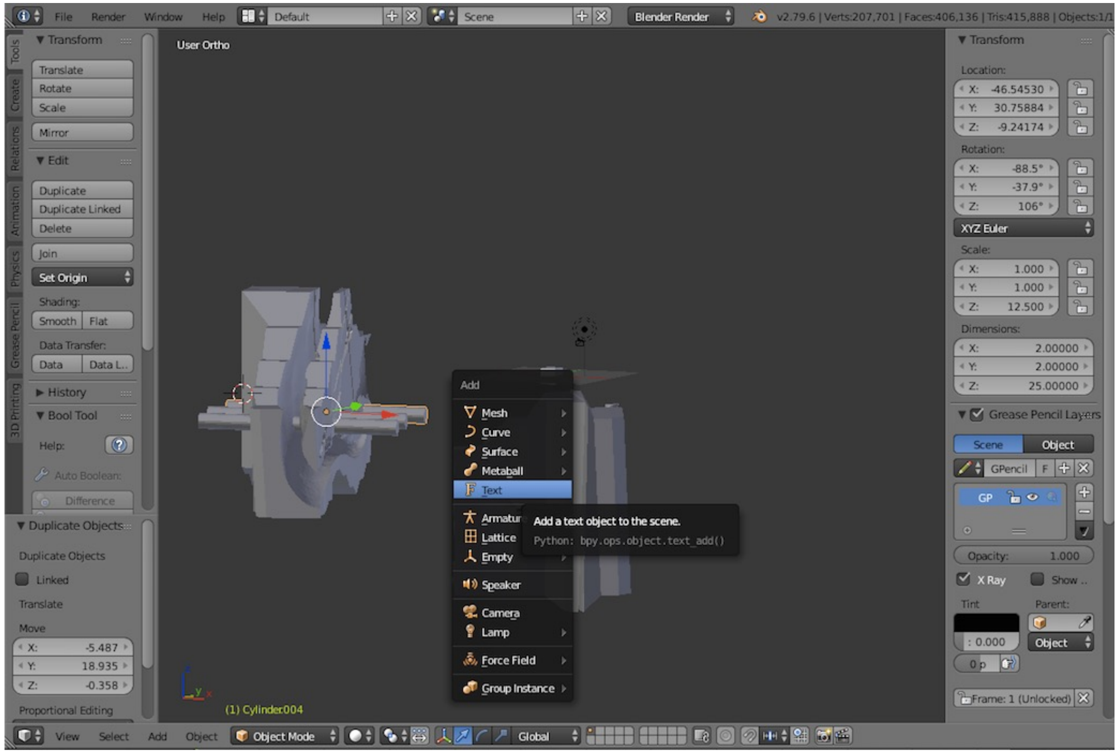

Cutting guides – Labelling

Blender
Version 2.79 (2.79 2018-09-10, Blender Foundation)

- Shift+A – Text
- *Edit Mode:* Modify text
- Extrude
- Position
- Transform Text into Mesh:
  - Alt+C – *Curves intro Mesh*
- Boolean: Difference guide-text mesh

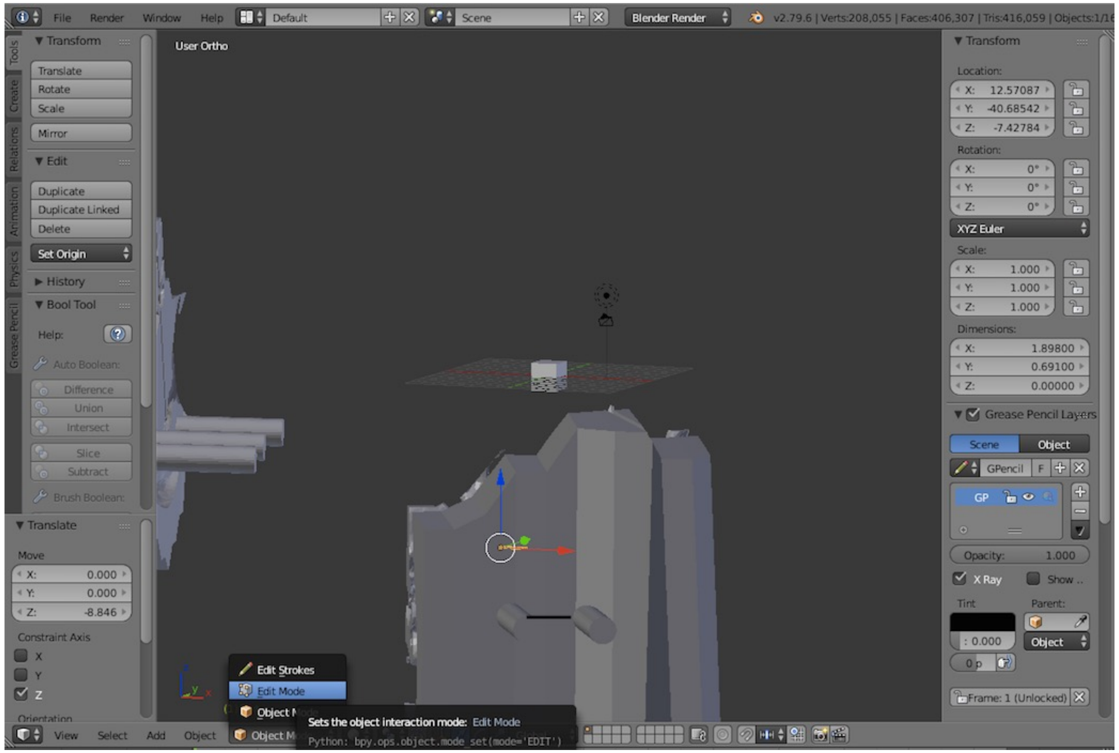

Cutting guides – Labelling

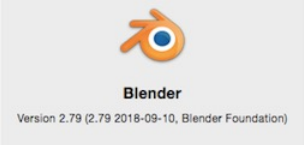

- Shift+A – Text
- *Edit Mode:* Modify text
- Extrude
- Position
- Transform Text into Mesh:
  - Alt+C – *Curves intro Mesh*
- Boolean: Difference guide-text mesh

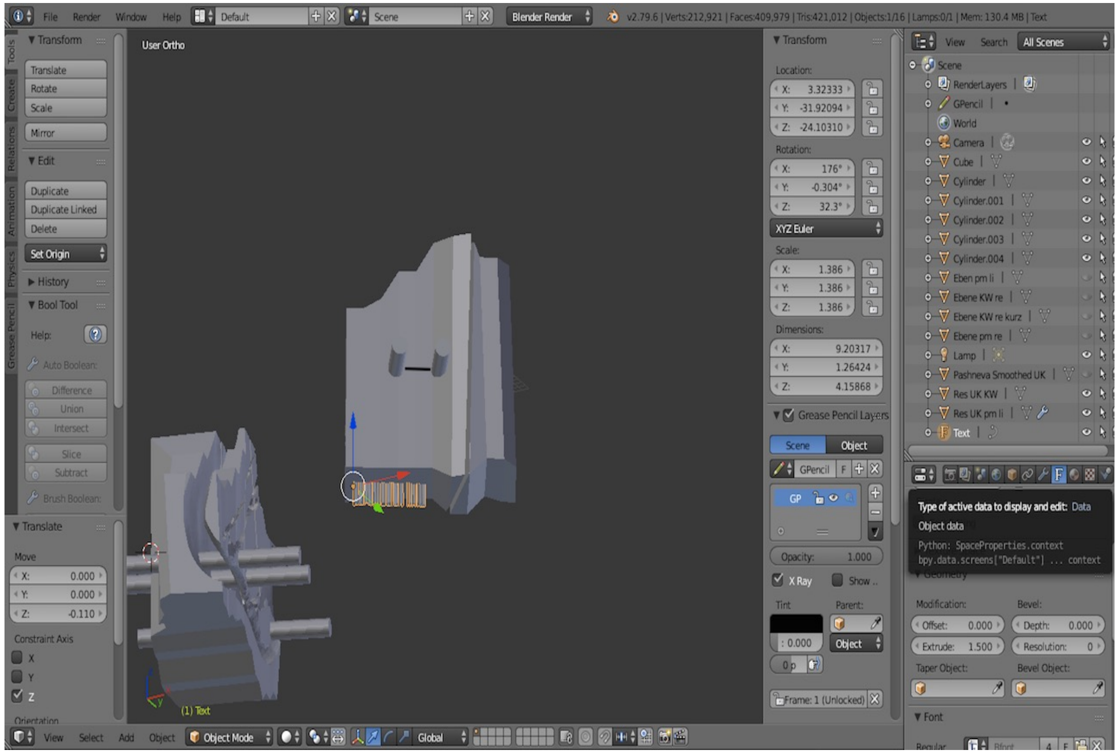

Cutting guides – Labelling

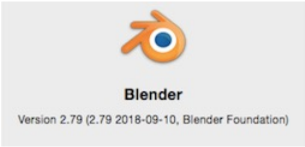

- Shift+A – Text
- *Edit Mode:* Modify text
- Extrude
- Position
- Transform Text into Mesh:
  - Alt+C – *Curves intro Mesh*
- Boolean: Difference guide-text mesh

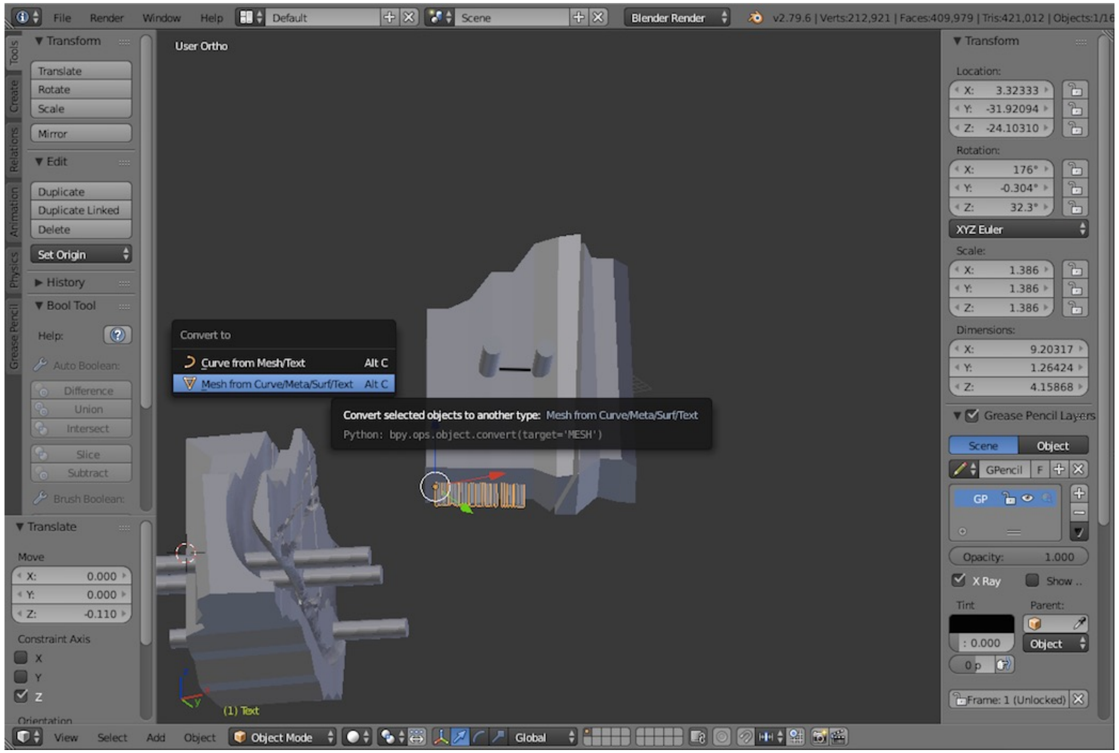

Cutting guides – Labelling

Blender
Version 2.79 (2.79 2018-09-10, Blender Foundation)

- Shift+A – Text
- *Edit Mode:* Modify text
- Extrude
- Position
- Transform Text into Mesh:
  - Alt+C – *Curves intro Mesh*
- Boolean: Difference guide-text mesh

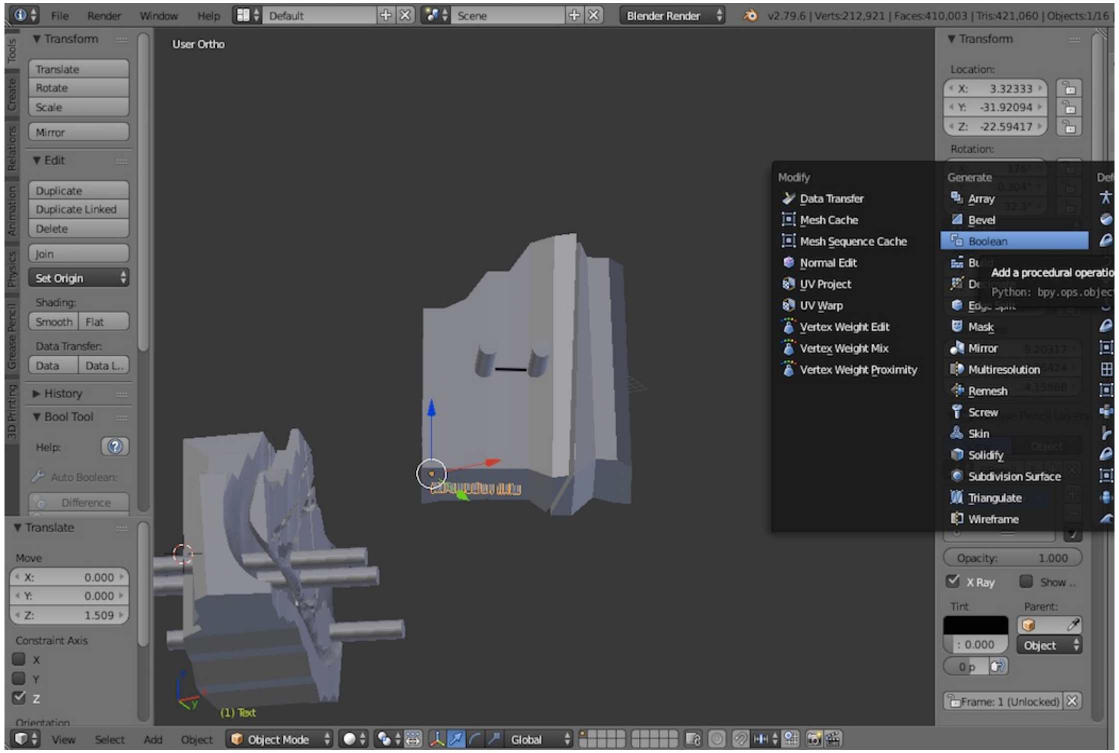

Supplement: Supplementary file 2 [file DataSheet_2.pdf]
